# Correction to: S08-2: Challenges of cooperation between urban planning, sustainable mobility, sport, and public health for effective HEPA policies - lessons from Slovenia

**DOI:** 10.1093/eurpub/ckaf133

**Published:** 2025-08-19

This is a correction to: Ina Šuklje Erjavec, Jana Kozamernik, Vita Žlender, S08-2: Challenges of cooperation between urban planning, sustainable mobility, sport, and public health for effective HEPA policies - lessons from Slovenia, *European Journal of Public Health*, Volume 34, Issue Supplement_2, September 2024, https://doi.org/10.1093/eurpub/ckae114.233

Originally, the incorrect article was published with this author list and title. This is emended, and they are now published with the correct article.

